# ABA signaling branches out: emerging ABA-related signaling functions in *Solanum tuberosum*

**DOI:** 10.1093/jxb/erad395

**Published:** 2023-11-21

**Authors:** José A Abelenda, Javier Barrero-Gil

**Affiliations:** Centro de Biotecnología y Genómica de Plantas, Universidad Politécnica de Madrid (UPM) – Instituto Nacional de Investigación y Tecnología Agraria y Alimentaria (INIA)/CSIC, Campus Montegancedo UPM, Pozuelo de Alarcón (Madrid), Madrid, Spain; Centro de Biotecnología y Genómica de Plantas, Universidad Politécnica de Madrid (UPM) – Instituto Nacional de Investigación y Tecnología Agraria y Alimentaria (INIA)/CSIC, Campus Montegancedo UPM, Pozuelo de Alarcón (Madrid), Madrid, Spain

**Keywords:** ABA signaling, axillary bud dormancy, drought tolerance, potato physiology, StHAB1

## Abstract

This article comments on:

**Liu T, Dong L, Wang E, Liu S, Cheng Y, Zhao J, Xu S, Liang Z, Ma H, Nie B, Song B**. 2023. StHAB1, a negative regulatory factor in abscisic acid signaling, plays crucial roles in potato drought tolerance and shoot branching. Journal of Experimental Botany **74**, 6708–6721.

**Despite its prominent role as a stress hormone and its related importance for agriculture, abscisic acid (ABA) molecular signaling is poorly characterized in crops such as potato (*Solanum tuberosum*). Potato is the world’s third most produced food crop and the most prominent non-cereal in terms of production and consumption (** **FAOSTAT, 2021** **). Its importance as a staple food is increasing worldwide. With the immediate need for stress-tolerant and more resilient plants due to changing climate patterns, research on ABA functions in potato will be essential to maintain productivity. Moreover, multiple developmental and physiological aspects of ABA unrelated to abiotic stresses are often overlooked and are difficult to study in plant models. Investigating putative ABA signaling players in potato plants, Liu *et al*. (2023) have deciphered the negative role of the phosphatase StHAB1 on ABA perception and potato drought stress responses. In addition, the authors provide conclusive evidence that ABA signaling is required for maintaining dormancy in aerial lateral buds. It has long been known that ABA stimulates tuber formation, but the molecular details of such mechanisms remain unknown. It was recently shown that the development of aerial lateral buds leads to a reduction in underground tuber production (Nicolas *et al.*, 2022). Therefore, the ABA-mediated blockage of lateral bud outgrowth reported by **Liu *et al*. (2023)** may provide a molecular explanation of the effects of ABA on tuber formation. The transgenic potato lines created in this study offer excellent genetic tools for further study of these connections and have potential biotechnological implications. This work underscores the importance of translational research from models to crops and how it is key not to circumscribe observations to obvious outputs in functional analysis in non-model plants.**

## Conserved roles of StHAB1 PP2CA phosphatase type in ABA signaling

In the model plant Arabidopsis, ABA synthesis is triggered under stress conditions such as drought, to be rapidly transported and perceived. The ABA signaling pathway relies on the interaction of a triple complex comprising a PYR/PYL/RCAR family receptor, protein phosphatases 2C (PP2Cs), and SNF1-Related Protein Kinase 2 protein kinases/SnRK2 (Santiago *et al.*, 2009; Nishimura *et al.*, 2010). In the absence of ABA, PP2Cs interact with SnRK2s, dephosphorylating them and preventing their kinase activity. ABA binds to the PYR/PYL/RCAR receptors and attaches them to PP2Cs, displacing the interaction away from SnRK2s and releasing their kinase activity to activate downstream effectors through protein phosphorylation (de Zelicourt *et al.*, 2016). Activated effectors, such as the transcription factors ABA-binding factors (ABFs), trigger the transcription of stress-responsive genes through binding to ABA-responsive elements (ABREs) in their promoters.

The ABA biosynthesis genes and signaling players are conserved in all seed plants, and seem to be present even in ferns (Cai *et al.*, 2017). Despite its importance, until recently, ABA was not described as an early drought stress signal regulating potato physiology (Liu *et al.*, 2005). Tomato (*Solanum lycopersicum*), a species closely related to potato, has been used as a model to study the functional antagonism between PYL/RCAR and the PP2Cs (González-Guzmán *et al.*, 2014), illustrating that the evolutionary conservation of the ABA signaling pathway allows direct biotechnological applications. The paper by Liu *et al*. (2023) addresses significant gaps regarding the molecular mechanisms of ABA signaling in *S. tuberosum*. It also provides conclusive evidence about the role of ABA in potato axillary bud dormancy and develops genetic tools that will be invaluable for studying the role of ABA in tuberization.

**Fig. 1.**
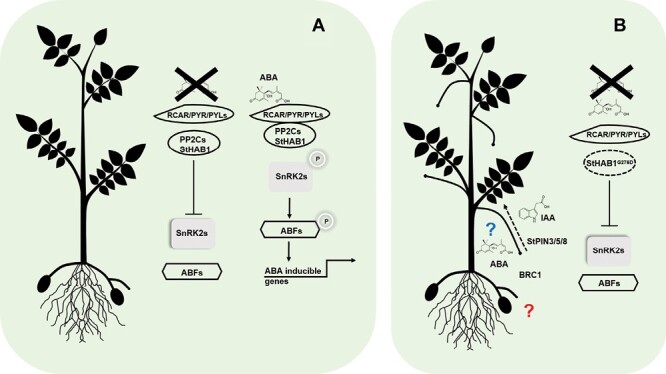
Model of the ABA signaling pathway in potato and putative roles related to branching and tuberization. (A) In *S. tuberosum*, the ABA core signaling pathway works with the same players as described in Arabidopsis and other plants. In the absence of ABA, RCAR/PYR/PYL receptors do not interact with PP2Cs/HABs. PP2Cs are free and active to bind and inhibit SnRK2s by dephosphorylation. ABA binding induces a conformational change in PYR/PYL receptors, which causes them to interact with PP2Cs, leading to the formation of an inactive complex of the receptor, ABA, and phosphatase. The inhibition of PP2Cs activates SnRK2 autophosphorylation and kinase activity, phosphorylating downstream effectors to induce the transcription of ABA response genes. (B) Dominant negative protein overexpression of the mutated *StHAB1*^*G276D*^ renders interaction with the RCAR/PYR/PYL receptor impossible and, consequently, results in the constitutive repression of SnRK2s kinases. The mutant presents developmental phenotypes besides ABA insensitivity related to drought stress, such as lateral bud outgrowth. The expression of the negative regulator *BRC1* is not altered in the mutant, but the auxin transporter genes *StPIN3/5/8* are up-regulated, pointing to a possible linear connection between BRC1, ABA perception, and IAA transport. Regardless of the phenotype, the causality of the relationship between the players in lateral bud development has not been mechanistically demonstrated (blue question mark). Stolons (belowground stems with the capacity to produce tubers) are strongly affected by ABA and auxins. Thus, it can be speculated that stolon development, maintenance, and tuberization will be altered in ABA-related mutants (red question mark).

In a classical approach, the authors first searched for the potato gene most closely related to the well-characterized Arabidopsis *PP2C HAB1* gene, called *StHAB1*. Several molecular experiments support the hypothesis that *StHAB1* has the same function as *HAB1*. The authors performed a consistent and valuable approach by looking at the phenotypes and physiological responses to abiotic stress of *StHAB1*-silenced and dominant overexpressor lines. These results confirmed the role of *StHAB1* as a negative regulator of ABA signaling: *StHAB1* knockdown transgenic plants are drought tolerant, whereas overexpression of a dominant negative version of the protein results in higher stress sensitivity. Moreover, the molecular signaling mechanism of HAB1 interacting with PYLs and SnRK2s is conserved in *S. tuberosum* (Fig. 1), and the authors demonstrate the interaction of these three elements using several independent techniques.

## Emergent roles of ABA in *S. tuberosum*

Validation experiments performed in species other than model systems, including crop species, are of great importance. From the point of view of translational science, considering a model plant as the gold standard to extrapolate concepts and molecular mode-of-action mechanisms is not always straightforward (Borrill, 2020). For instance, the developmental program and reproductive habits of *S. tuberosum* are not comparable to those of *Arabidopsis thaliana*. Potato plants have two independent but coordinated reproductive systems: they can reproduce sexually by producing flowers and seeds, and vegetatively through the formation of tubers (Plantenga *et al.*, 2018). In addition, the plant architecture of potato is not monopodial like that of Arabidopsis but sympodial, allowing for lateral branches with non-consecutive levels of development after the termination of the main stems into inflorescences (Kacheyo *et al.*, 2021). Altogether, this may imply the co-option of new regulators such as ABA at the developmental level.

The authors found that potato transgenic lines overexpressing a dominant mutated version of *StHAB1* presented extreme lateral bud outgrowth (Fig. 1). The master negative regulator of axillary bud outgrowth in Arabidopsis is *BRANCHED1* (*BRC1*), whereas in potato it is *BRANCHED1a* (*BRC1a*) (Nicolas *et al.*, 2015). Moreover, a different potato *BRC1* paralog, *StBRC1b*, positively affects underground tuberization (Nicolas *et al.*, 2022), avoiding the formation of aerial tubers. In Arabidopsis, it has been shown that *BRC1* induces local ABA synthesis, which is essential for lateral bud growth suppression (González Grandio *et al.*, 2016). If lateral bud dormancy is necessary for tuber formation, it is tempting to speculate that the positive effects of ABA on potato tuberization (Muñiz García *et al.*, 2014) are partially achieved through the suppression of lateral bud outgrowth in a *BRC1*-dependent manner. Although the authors observe that alteration of ABA signaling does not affect *BRC1* expression, it might well be that a *BRC1* homolog works upstream of ABA signaling in the same fashion as in Arabidopsis. Interestingly, the expression of the auxin transporter genes *StPIN3*, *StPIN5*, and *StPIN8* increases in the transgenic plants, remobilizing auxin from the axillary buds, which indicates a connection between ABA and auxin signaling.

## Research and translational prospects

Potato is an Andean crop, well adapted to cool and temperate environments. However, drought and rising temperatures affect its productivity and survival more compared with other crops due to the insufficient capacity of potato plants to absorb and retain water from the soil because of their shallow root system (Weisz *et al.*, 1994). With extreme climatic conditions likely to become more frequent in the coming years and the need to increase plant resilience against adverse conditions, a comprehensive description of the leading players involved in abiotic responses in several crops will be paramount. Orthogonal and chemical treatments may enhance plant drought tolerance, manipulating the ABA response at the molecular level (Helander *et al.*, 2016). Nevertheless, different species will probably need tailor-made specific treatments. Such approaches, and knowledge about molecular aspects of ABA perception and signaling, will be vital in the future.

Tuberization is a complex trait that is dependent on the formation and growth of modified belowground lateral stems called stolons. Since stolons are lateral shoots, the transgenic dominant *StHAB1* lines could also show changes in stolon number, development, and progression until tuber formation. Drought severely impacts tuber formation, altering carbon source–sink relationships between different plant organs (Aliche *et al.*, 2020), and ABA signaling likely plays a central role in these processes. Indeed, there is a clear connection between the induction of tuberization and the establishment of new strong sinks (Nicolas *et al.*, 2022). A deeper investigation of these aspects will open paths to understanding stolon development and how it progresses in both well-watered and drought conditions. Finally, the connection between ABA and auxin highlighted in this paper is of great interest, considering the critical role of auxin during tuberization and tuber development (Roumeliotis *et al.*, 2012, 2023). We have poor insights about the cross-talk of strigolactones/auxin and ABA in stolons and how they affect tuberization. Phenotyping and molecular characterization of relevant mutants, together with targeted molecular approaches, will help us understand traits that are critical for the optimization of yield and plant resilience.

## Conflict of interest

No conflict of interest declared.
